# Supplementary material for: predicTTE: An accessible and optimal tool for time-to-event prediction in neurological diseases
Source: bioRxiv. 2024 Jul 23:2024.07.20.604416. Preprint. [Version 1] doi: 10.1101/2024.07.20.604416 (PMC11291041; doi:10.1101/2024.07.20.604416)
Supplement: 1 [file NIHPP2024.07.20.604416V1-supplement-1.pdf]

bioRxiv preprint doi: https://doi.org/10.1101/2024.07.20.604416; this version posted July 23, 2024. The copyright holder for this preprint (which was not certified by peer review) is the author/funder, who has granted bioRxiv a license to display the preprint in perpetuity. It is made available under aCC-BY 4.0 International license.

Supplementary Material Legends

Supplementary Table 1: Hyperparameters for optimal prediction models in each use-case.

Supplementary Table 2: Prediction model performance for use-case 1 including randomised training data, and with/without imputation which included/did not include the outcome variable. Model performance is measured by the difference, absolute difference, absolute difference

bioRxiv preprint doi: https://doi.org/10.1101/2024.07.20.604416; this version posted July 23, 2024. The copyright holder for this preprint (which was not certified by peer review) is the author/funder, who has granted bioRxiv a license to display the preprint in perpetuity. It is made available under aCC-BY 4.0 International license.

normalised for patient survival and C-index. We also show the proportion of predictions within 'x' years and % of actual patient survival. DL = optimum deep learning model. RP = Royston-Parmar which was the optimum spline model.

Supplementary Table 3: Number (%) of missing data points for use-case 1

Supplementary Table 4: Prediction model performance for use-case 2. Model performance is measured by the difference, absolute difference, absolute difference normalised for patient survival and C-index. We also show the proportion of predictions within 'x' years and % of actual patient survival.

Supplementary Table 5: Prediction model performance for use-case 3. Model performance is measured by the difference, absolute difference, absolute difference normalised for patient survival and C-index. We also show the proportion of predictions within 'x' years and % of actual patient survival.

Supplementary Table 6: Example patients where choice of anticoagulation with either dabigatran, rivaroxaban, or apixaban impacts survival time. Corresponding to **Fig. 5d.**

Supplementary Video 1: Demonstration of the predicTTE online portal for model training, individualised prediction and data sharing

bioRxiv preprint doi: https://doi.org/10.1101/2024.07.20.604416; this version posted July 23, 2024. The copyright holder for this preprint (which was not certified by peer review) is the author/funder, who has granted bioRxiv a license to display the preprint in perpetuity. It is made available under aCC-BY 4.0 International license.

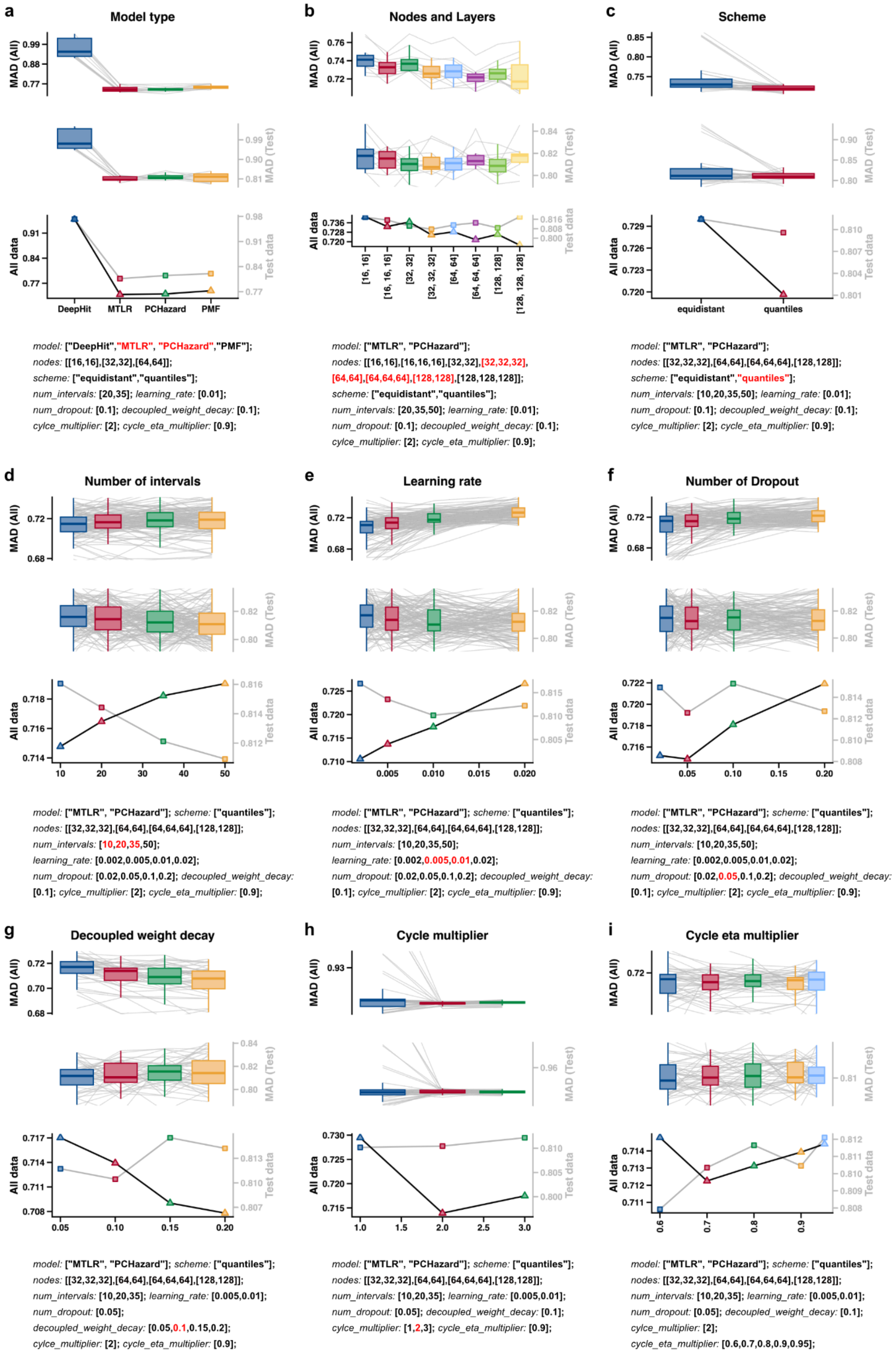

bioRxiv preprint doi: https://doi.org/10.1101/2024.07.20.604416; this version posted July 23, 2024. The copyright holder for this preprint (which was not certified by peer review) is the author/funder, who has granted bioRxiv a license to display the preprint in perpetuity. It is made available under aCC-BY 4.0 International license.

Supplementary Figure 1: Hyperparameter tuning and model choice. The figure illustrates the step-by-step process of hyperparameter tuning. Plotted data denotes prediction performance derived from every tested combination of hyperparameters. Each panel demonstrates the effect of varying a single hyperparameter using the training data from use-case 1. The specific hyperparameter is noted in the title; boxplots are shown for the median absolute difference between actual and predicted time to event (MAD) in the entire training dataset (top subpanel), and in the external validation dataset (middle subpanel). For comparison, in the bottom subpanel the MAD value is shown for both the entire cohort (black line) and the external validation dataset (grey line). To avoid testing all possible combinations of hyperparameters, which could lead to overfitting, we select hyperparameters in a series of discrete steps. The first step includes hyperparameters which are more likely to have a large effect on prediction performance. In the first step only the *model type* was tested (**a**); in the second step the possible *nodes and layers* were reduced to a smaller subset and within the same models the *scheme* was selected (**b, c**). In the third step the *number of intervals*, the *learning rate* and the *number of dropout* were selected from the same model combinations (**d, e, f**). Finally the *decoupled weight decay* (**g**), *cycle multiplier* (**h**) and *cycle eta multiplier* (**i**) were chosen. Underneath each panel there is a list of all hyperparameters tested; red hyperparameters in a specific category are those taken forward to the next step of hyperparameter tuning whereas black hyperparameters in that same category are dismissed. The interpretation of hyperparameters is found in[3]. Other hyperparameters including the number of epochs and batch size had a minimal effect on prediction performance (data not shown).

bioRxiv preprint doi: https://doi.org/10.1101/2024.07.20.604416; this version posted July 23, 2024. The copyright holder for this preprint (which was not certified by peer review) is the author/funder, who has granted bioRxiv a license to display the preprint in perpetuity. It is made available under aCC-BY 4.0 International license.

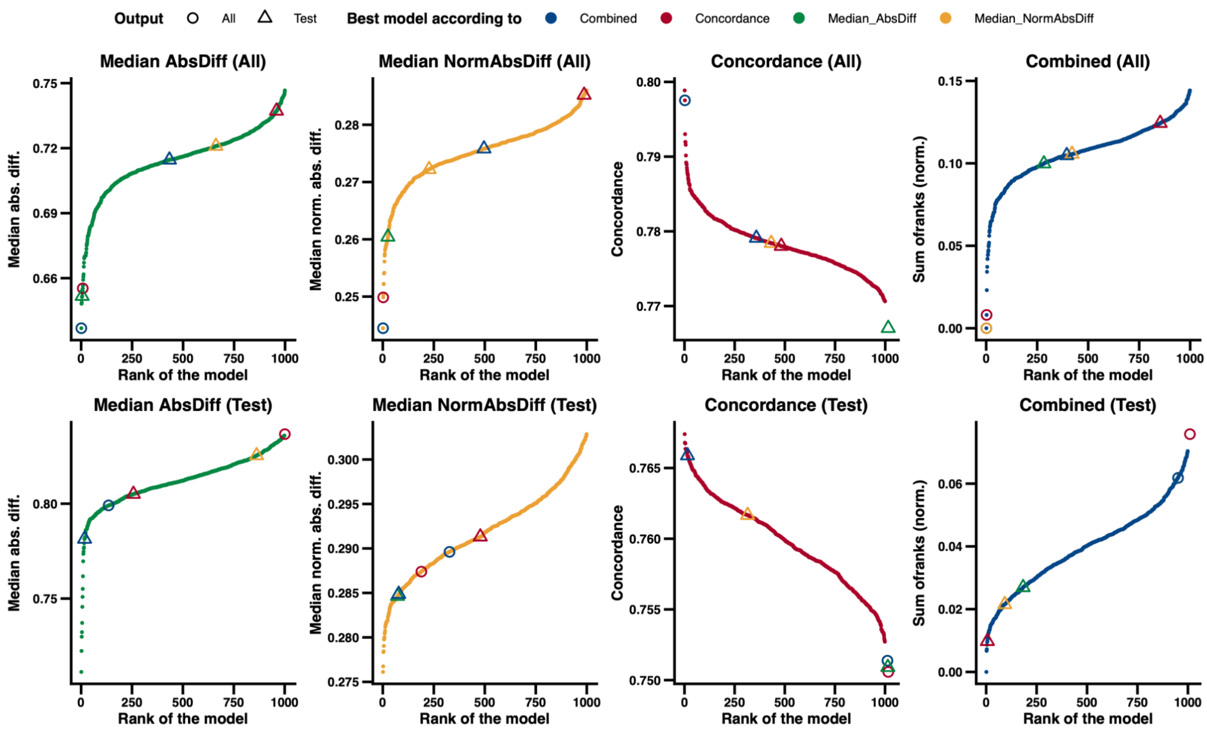

Supplementary Figure 2: Ranking of models during hyperparameter tuning. This figure illustrates the concept behind choice of the optimal model via hyperparameter tuning. Model rank and performance relates to the best 1000 models tested during hyperparameter tuning for use-case 1, as described in **Supplementary Fig. 1**. The top row shows model rank and performance in the entire training set (60% training, 20% validation, 20% external validation) and the bottom row shows model rank and performance for the 20% external validation set. From left to right the outcome measurements: median absolute difference, normalised median absolute difference (median absolute difference divided by observed time), concordance and combined (normalised sum of the ranks of all three outcome measurements) are shown and coloured differently. For comparison, in each plot the rank of the best model according to a *different outcome measure* is shown in the corresponding colour, and with circles for the entire data and triangles for the external validation data. As demonstrated the best combined (summed rank for all outcome measures) model for the entire dataset (that was picked as the optimal model) performs well (is ranked low) in all other outcome measurements (blue circle) apart from the concordance in the external validation dataset, although the difference to the best model was low (0.015). On the other hand picking the model that performs best in the external validation set with respect to concordance (red triangle) leads to a relatively poor performance would be low in all other measurements.

bioRxiv preprint doi: https://doi.org/10.1101/2024.07.20.604416; this version posted July 23, 2024. The copyright holder for this preprint (which was not certified by peer review) is the author/funder, who has granted bioRxiv a license to display the preprint in perpetuity. It is made available under aCC-BY 4.0 International license.

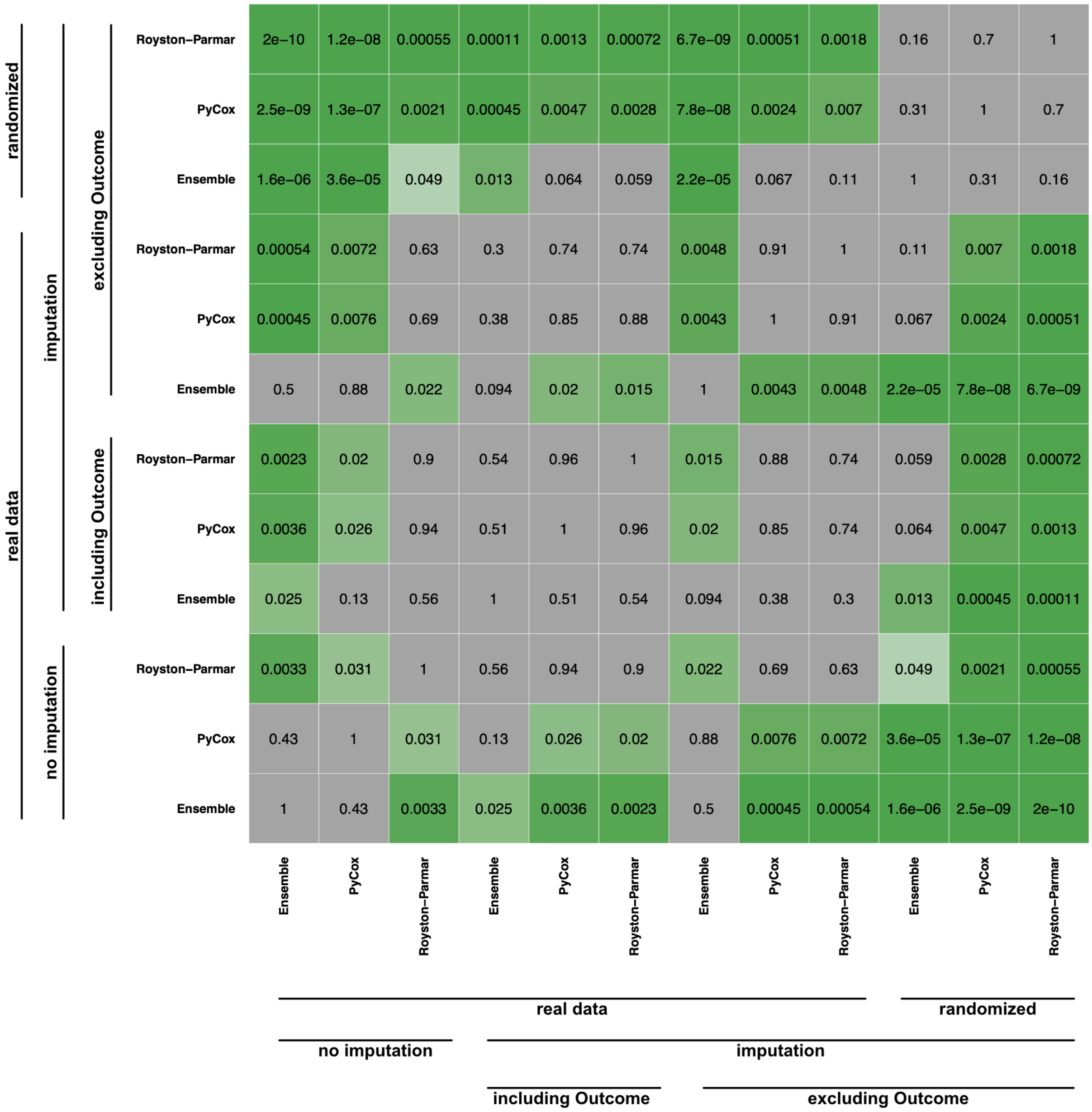

Supplementary Figure 3: Prediction model performance for use-case 1 including shuffled training data, and with/without imputation which included/did not include the outcome variable. P-values are shown for a Wilcoxon rank-sum test comparing absolute difference between predicted and observed survival for each pair of models. Pycox = optimum deep learning model. Royston-Parmar = the optimum spline model. Ensemble indicates the ensemble model including a second stage of focused model training. The lowest median absolute difference between predicted and observed survival for a model which included imputation, was achieved with the ensemble model, with imputation which did not include the outcome variable (**Fig. 3d**); the difference between performance of this model and alternatives was statistically significant ($p<0.05$) with the exception of the ensemble model with imputation which *did* include the outcome variable ($p=0.094$).
